# “An Unusual Urological Tumour”: Above the Collar and below the Belt

**DOI:** 10.1155/2012/480826

**Published:** 2012-09-29

**Authors:** Erik K. Mayer, Shabnam Undre, Daniel C. Cohen, Marjorie M. Walker, Justin A. Vale, Anup Patel

**Affiliations:** ^1^Department of Urology, St. Mary's Hospital, Imperial College Healthcare NHS Trust, Praed Street, London W2 1NY, UK; ^2^Department of Histopathology, St. Mary's Hospital, Imperial College Healthcare NHS Trust, Praed Street, London W2 1NY, UK

## Abstract

Bladder lymphomas are rarely primary tumours and more commonly associated with systemic lymphoma, either as nonlocalised bladder lymphoma or as secondary bladder lymphoma. Primary bladder lymphomas (PBL) tend to be low-grade mucosa-associated lymphoid tissue (MALT) type, contrasting with diffuse large cell or follicular centre cell types more commonly seen in secondary bladder lymphoma. Bladder involvement by systemic lymphoma infers poor prognosis and patients often have no localising symptoms (typically a postmortem diagnosis). Other treatments are preferred over surgery for all bladder lymphomas, except where diagnosis is uncertain or for relief of irritative bladder symptoms. We describe a unique case of systemic high-grade B-cell lymphoma with simultaneous cutaneous renal and bladder lesions at presentation.

## 1. Case Report

**Figure 1:**
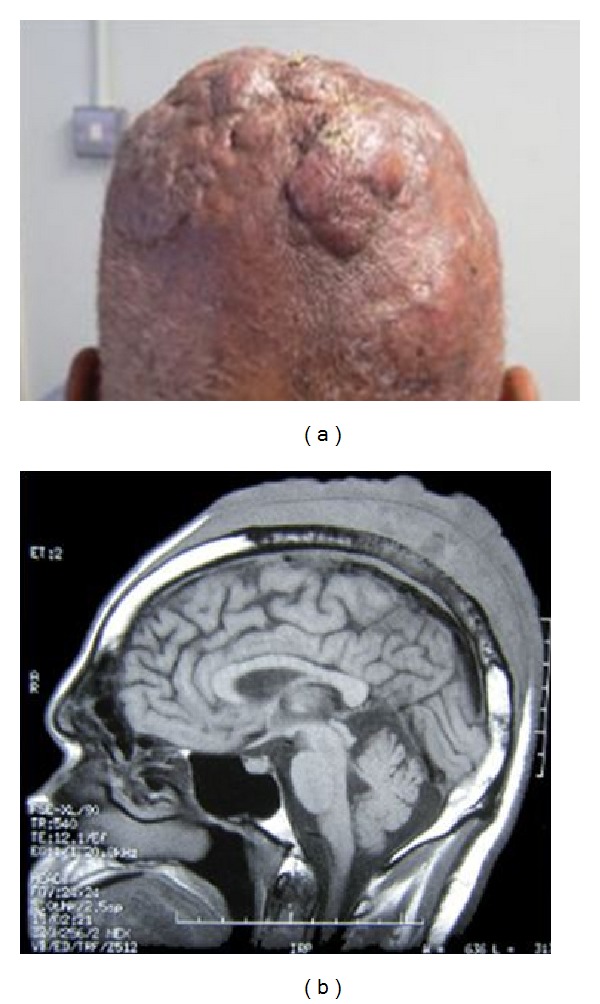
(a) Scalp nodules as seen at presentation. (b) MRI demonstrating an extensive mixed signal, soft tissue mass, over the vertex of the skull. There is infiltration through the inner and outer tables of the skull vault and extension into the dural membranes.

A 70-year-old man presented to the dermatologists with a 2-year history of painless enlarging scalp nodules, which had rapidly worsened over the previous 6 months (Figure 1(a)). He had lost 7 kg in weight; there were no other systemic symptoms. He had neither clinical lymphadenopathy nor hepatosplenomegaly and his peripheral blood count, ESR, protein electrophoresis, liver function tests, and LDH were normal. A skin biopsy from the scalp demonstrated high-grade B-cell non-Hodgkin's lymphoma. Staging CT showed multiple small cervical lymphadenopathy, lymphomatous infiltration in both kidneys, and an area of mucosal thickening of the bladder, which was consistent with a primary bladder carcinoma. Staging was completed with an MRI of the head (Figure 1(b)) and bone marrow biopsy, which was normal. 

**Figure 2:**
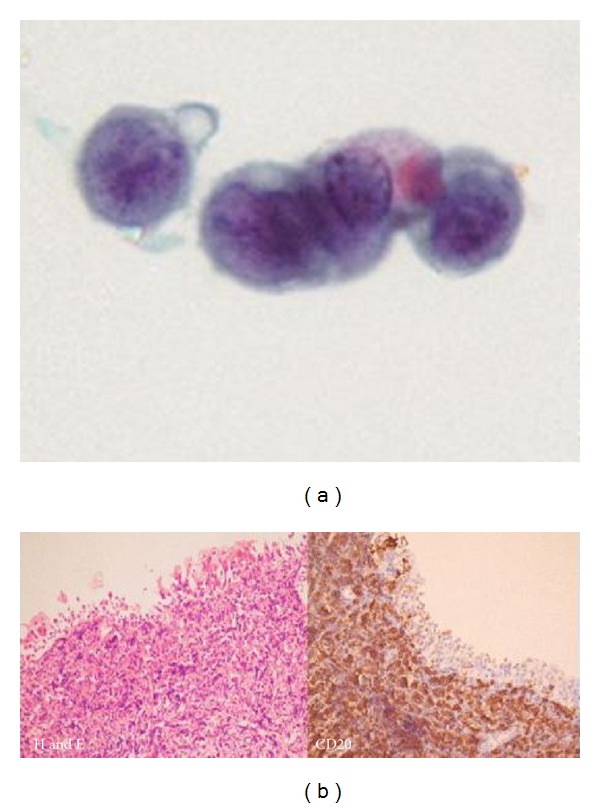
(a) Urine cytology demonstrates a cluster of atypical lymphoid cells. (b) Biopsy of bladder tumour showing infiltration of the transitional epithelium and lamina propria by atypical B lymphocytes (CD20 positive on immunohistochemistry).

The patient described neither lower urinary tract symptoms nor episodes of macroscopic haematuria and was a nonsmoker. A urine cytology specimen revealed atypical cells more consistent with lymphoid than epithelial/transitional cell origin (Figure 2(a)). At cystoscopy, a 3 cm round submucosal lesion on the anterior aspect of the bladder wall was seen and resected. Histology confirmed high-grade B-cell lymphoma in both bladder and kidney biopsies (Figure 2(b)).

## 2. Discussion

To date there are few case reports of nonprimary bladder lymphoma, reflecting its rarity as a presenting pathological entity. To our knowledge we describe the first reported case of disseminated lymphoma with simultaneous cutaneous, renal, and bladder lesions demonstrable at presentation.

The incidence of non-Hodgkin's lymphoma (NHL) is increasing and maintains a geographical variability: in 2007 there were 10,900 people diagnosed with NHL in the UK [1]. The incidence of NHL typically increases with age. Rates increase sharply in people over 50 and around two-thirds (68%) of all cases are diagnosed in people over 60 years. The most recent classification system is that endorsed by the World Health Organisation (REAL classification) and includes subtypes such as diffuse large B-cell, follicular, and small lymphocytic. Determinations of specific subtypes of disease along with patient's prognostic characteristics gleaned from the International Prognostic Index are crucial to treatment plans and subsequent management. 

Lymphomas of the bladder are rare and account for 0.2% of all bladder neoplasms [2]. There is a female predominance and average age of presentation of 58 years. The most common type is extranodal marginal zone lymphoma of mucosal-associated lymphoid tissue (MALT) first described in 1990 [3], while Hodgkin's type is even rarer. Lymphomas are classed into the primary group if they present primarily with bladder symptoms and there is no evidence of systemic lymphoma at presentation. 

More commonly, although relatively few case studies exist, lymphoma of the bladder is seen in association with systemic lymphoma; this can either be as bladder involvement with active systemic lymphoma (nonlocalised bladder lymphoma), as reported in this case study, or as lymphoma recurrence in the bladder after a period of remission from previous lymphoma (secondary lymphoma) [3]. It is rare for disseminated lymphoma to present with symptoms of bladder involvement and more typically remains a postmortem diagnosis [2]. Although a percentage of patients with bladder involvement experience no urinary symptoms, urinary urgency, frequency, and haematuria have all been described [3]. It has been reported that a high proportion (47%) of patients in this group experience ureteric obstruction [3].

Nonprimary bladder lymphomas tend to be a different histological type than those found in the PBL group and are commonly of the diffuse large cell or follicular centre cell types [3]. In the nonprimary group, the bladder involvement by systemic lymphoma infers a poorer prognosis than that seen in PBL as it implies multisystem disease. However, there is a clear prognostic distinction between the secondary bladder lymphoma and non-localised lymphoma groups with median survival of 0.6 years and 9 years, respectively [3]. Part of this might be explained by some patients being classified in the non-localised group when a dominant bladder mass was only associated with local extension to adjacent organs or to regional lymph nodes [3]. The authors argued that although some might classify this category of patient into a “PBL” group, their tumour histology resembled more closely that of the general population and the patient and disease characteristics were distinct from those seen in the PBL group [3]. 

Non-Hodgkin's lymphoma involving the bladder can be successfully treated using chemotherapy, radiotherapy, immunotherapy, surgery, and combinations of these modalities [4]. The treatment of choice is determined by the histology and extent of disease. The systemic nature of nonprimary bladder lymphoma warrants a systemic approach to treatment. Lymphomas with aggressive phenotypes such as diffuse large B-cell are best managed with chemotherapy. The cyclophosphamide, doxorubicin, vincristine, prednisone, and rituximab (CHOP/R) regime is most commonly used [4]. Adjuvant radiotherapy is used for involved fields in localised disease. Follicular lymphoma treatments range from a watch-and-wait policy to chemoimmunotherapy or radioimmunotherapy. Immunotherapy generally forms a standard component of management of advanced disease [4].

The indolent nature of the MALT lymphomas means that local radiotherapy can be used with curative intent, such as in PBL. Surgical resection has a limited role in primary bladder lymphoma, beyond obtaining biopsy specimens, and rarely adds anything to management or prognosis. There are reports, however, of patients having undergone cystectomy due to unclear diagnosis, or to relieve irritative symptoms [5]. There is a report of a low-grade MALT-type PBL being managed with transurethral resection and adjuvant intravesical mitoxantrone [6].

In this case, the patient's disease constituted stage IV B. He underwent eight cycles of CHOP/R chemotherapy followed by radical radiotherapy to scalp and brain. Follow-up MRI showed dramatic improvement in the scalp and dural mass with minimal residual dural enhancement. A repeat CT showed complete remission with no residual disease in either kidneys or bladder. The patient remained in complete remission for 14 months but then developed a gradual deterioration in his balance and thereafter a marked peripheral neuropathy. Repeat imaging confirmed recurrent widespread cerebral metastasis. He died following a cardiac arrest before palliative radiotherapy could be given.
